# Supplementary material for: All-at-once RNA folding with 3D motif prediction framed by evolutionary information
Source: Nat Methods. 2025 Oct 3;22(10):2094–106. doi: 10.1038/s41592-025-02833-w (PMC12510878; doi:10.1038/s41592-025-02833-w)
Supplement: Supplementary file 3 — Source code to run CaCoFold-R3D. Data and results. [file 41592_2025_2833_MOESM3_ESM.gz › supplemental_material/results/CaCoFold-R3D/CaCoFold-R3D_Rfam_vshuffle/RF01880_Xist_exon1.R2R.sto.pdf]

RF01880_Xist_exon1

5′ - A A A U C A A A A U C C R A C C C C A G C A U U A G C C A Y G G G G U A G R U G ● U C C U ● U ● G A R G A A G G Y A G G ○ A U U C C ● C Y U C U G C Y A ● ● ● ● ● U ● R C
